# A Physiological and Kinematic Comparison of two Different Lean Back Positions During Stationary Rowing on a Concept II Machine

**DOI:** 10.2478/hukin-2013-0030

**Published:** 2013-07-05

**Authors:** Gordon Bell, Jack Bennett, William Reynolds, Daniel Syrotuik, Pierre Gervais

**Affiliations:** 1 Faculty of Physical Education and Recreation, University of Alberta, Edmonton, Alberta, Canada.

**Keywords:** oxygen uptake, efficiency, power output, joint angle, range of motion, rowing

## Abstract

This study compared two different body positions at the finish of a stroke during stationary rowing exercise on physiological and kinematic measurements. Nine male and five female rowers volunteered for the study: mean age (± SD), body height and body mass were 27 ±9 yrs, 180.5 ±12.3 cm and 81.2 ±14.2 kg. The two body positions at the finish were controlled at an upright posture or a novel greater lean back position. All subjects completed 3 different experimental trials on a Concept IID rowing machine at 3 different exercise intensities and comparisons were made between the lean back position at the same stroke rate and the same power output as the upright trial. Power output, heart rate, oxygen uptake, energy expenditure and % efficiency were higher (p<0.05) with the greater lean back position at the same stroke rate compared to all other conditions. Range of motion at the hip, ankle, and elbow and the handle velocity and distance moved were greater (p<0.05) with the lean back position. In conclusion, a greater lean back posture at the finish during stationary rowing produces a higher power output and improved efficiency at the same stroke rate but at an elevated physiological cost compared to a more upright position. Despite the higher energy expenditure, the relative gain in power output and efficiency with no negative kinematic changes suggests that a greater lean back position at the finish will enhance performance during stationary rowing exercise.

## Introduction

The effective transfer of force to the handle of a rowing machine during stationary rowing or to the oar of a racing shell on water requires appropriate technique to execute the mechanics of the rowing stroke ( Bompa, 1980 ; Martindale and Robertson, 1984 ; Torres-Moreno et al., 1999 ; Barrett and Manning, 2004 ; Webster et al., 2006 ). The phases of the rowing stroke begin in the “catch” position that is the start of the power development followed by a coordinated sequence of muscular actions about all the major joints of the body through the “drive” phase to the “finish” position of the stroke. The sequences of these events are then reversed (“recovery” phase) to return to the catch position to complete the rowing stroke ( Mazzone, 1988 ; Secher, 1993 ; Nolte, 2005 ; Webster et al., 2006 ). During the recovery phase, there is a deceleration of the rowing machine flywheel during stationary rowing and the racing shell velocity during on water rowing ( Millard, 1987 ). This basic sequence of phases are repeated at various stroke rates depending on the speed/power output desired; the pacing strategy used during a simulated or actual 2000 m rowing race; or, the stroke rate required for physical training or fitness testing of a rower ( Bell et al., 1989 ; Hartman et al., 1994 ; Torres-Moreno et al., 1999 ; Gillies and Bell, 2000; Ingham et al., 2002 ; Kennedy and Bell, 2003 ; Mandic et al., 2004 ; Garland, 2005; Webster et al., 2006 ; Volger et al., 2010 ). Despite obvious differences in the kinematics of rowing a racing shell on water (sweep or scull) and rowing a stationary ergometer, many physiological requirements of the rowing action ( Hagerman, 1984 ; Martindale and Robertson, 1984 ; Secher, 1993 ; Urhausen et al., 1993 ; Volger et al., 2010 ) and the basic sequences of movement patterns in a general rowing stroke are similar between the two modes ( Secher, 1993 ; Nolte, 2005 ; Webster et al., 2006 ).

Stationary rowing exercise has become popular for recreation, rehabilitation, cross training, competition and as adjunct to rowing on the water. Also, it is often prescribed by rowing coaches for on-land fitness training, to aid in seat selection of rowing crews and to determine rowing race performance and fitness off-water ( Klusiewicz et al., 1999 ; Gillies and Bell, 2000). In addition, there is an annual world indoor 2000 m rowing championship that includes categories for individuals with various disabilities ( C.R.A.S.H.-B Sprints, 2012 ). As a result of this popularity and competition, increasing interest in exploring differences in technique that can lead to an improvement in stationary rowing performance may be observed. One particular aspect of the rowing stroke that has been understudied is the effect of different lean back positions at the finish of the stroke. A direct consequence of a greater lean back position at the finish would be a longer stroke and potentially, a greater power output compared to a more upright body position. This would translate to a greater performance (increase distance rowed per stroke and improved time) during stationary rowing exercise. However, this modification would be more energy demanding as well due to the increase in muscular work to complete the more extended body position at the finish. Few studies have investigated the biomechanical aspects of stationary rowing ( Torres-Moreno et al., 1999 ; Webster et al., 2006 ) and there is no research that has systematically compared the “power output benefit to energy cost” of different lean back positions at the finish of the stroke during stationary rowing exercise despite the potential advantages. Therefore, the purpose of this study was to compare the physiological and kinematic responses to stationary rowing exercise of different intensities using two lean back positions at the finish. It was hypothesized that a greater lean back position at the finish would increase the range of motion about the hip, allowing for a longer rowing stroke that would be more energy demanding (e.g. higher oxygen uptake) but generate a greater power output when compared to rowing at a more upright position while rowing at the same stroke rate. The extent to which the potential benefit in power output production outweighed the energy cost was also explored under standard testing conditions.

## Material and Methods

### Participants

A convenient sample of 14 (9 male and 5 female) rowers volunteered for the study as a result of a poster advertisement and word of mouth. The mean age (SD), body height, body mass and V̇O _2_ peak was 25 ±4 yrs, 171.4 ±8.1 cm, 74.6 ±7.7 kg, 44.9 ±5.3 ml×kg ^−1^ ×min ^−1^ and 29 ±11 yrs, 185.6 ±11.5 cm, 84.8 ±15.9 kg and 51.5 ±6.9 ml×kg ^−1^ ×min ^−1^ for women and men, respectively. The sample included both light and heavy weight rowers and all were actively training for competition. The experience level ranged from 3 to several years of competitive rowing experience at a provincial or national level.

### Measures

Each subject arrived at the laboratory for the testing sessions after refraining from any formal exercise for 24 hours and consuming a light meal of their choice that was advised by the researchers to include complex carbohydrates and a small amount of fat and protein and water ad libitum 2 to 3 hours before each exercise test. The 2 to 3 hour time frame was implemented to reduce any influence of diet on the metabolic variables being examined. Body mass (kg) was measured on a balance beam scale and body height (cm) was measured using an anthropometric tape measure and a right angle plane placed on the head of each subject while standing shoeless against a wall.

Aerobic fitness was assessed with an incremental, peak oxygen uptake (V̇O _2_ peak ) test to volitional exhaustion on a Concept IID rowing machine. Ventilatory parameters and gas exchange variables were measured with a calibrated metabolic system (ParvoMed, Utah) and averaged every 20 s. Heart rate (HR) was determined each minute using a telemetric monitor (Polar Pacer, Finland). The exercise testing protocol and termination criteria for this assessment has been previously described in detail for both men and women ( Gillies and Bell, 2000).

On a separate day, each subject was required to perform the 3 different experimental exercise trials during the same session. Each trial involved 3 different submaximal exercise intensities that were 3 to 5 minutes in duration to ensure steady state V̇O _2_ was achieved (< 0.100 L×min ^−1^ change in V̇O _2_ over 1 minute of exercise ). Metabolic and heart rate data were continuously collected using the same methods described previously. The bouts were separated by a minimum of 5 minutes of non-exercise recovery and until HR was < 100 b×min ^−1^ . To control the extent of the LB position at the finish of the rowing stroke, a structure was built that included a padded bar placed horizontally on two stands. During exercise, each rower was required to LB until they touched the bar with the upper back (lower to mid scapula) on every stroke. A goniometer was used to set the angle of the hip at the finish to a minimum of 105° for the upright position and 150° for the LB finish. This angle was measured between the thigh and torso for the different experimental session. The hip angle was determined for each trial and subject using the goniometer and specific placement of a bar to impede the extent of LB at the finish. Modeling the trunk as a rigid segment could not take into account the different angular displacements due to shoulder retraction and upper trunk extension that resulted during actual physical trial conditions. As well, there was some flex and padding in the apparatus as a safety consideration. As a result, the mean hip angle for the upright body position used in Trial 1 was ∼117° and ∼145° for the greater LB position used in Trials 2 and 3. The participants were allowed to briefly practice the LB positions during each trial and verbal feedback was provided by the researchers to assist the participants in meeting the LB hip angle criteria during the experiments.

Biomechanical data was collected at the same time as the physiological measurements during each experimental trial. Light retro-reflective markers were attached to anatomically landmarked positions on the body, the rowing machine and stand using two sided tape ( Picture 1 ). Marker positions were monitored using 5 Qualisys ProReflex (Gothenburg, Sweden) motion tracking cameras operating at 60Hz. In addition to the infra-red motion tracking, a stationary video camera, whose optical axis was set perpendicular to the movement plane and synchronized with the Qualisys system, recorded each trial at a rate of 60 fields per second. The ankle, knee, hip and elbow angles as well as the distance and velocity that the handle moved from catch to finish was determined using custom software written in Matlab (Torrence, California).

### Procedures

Each participant attended an orientation meeting to receive an explanation of the purpose and procedure of the study, provide basic demographic information and be informed on the use of laboratory equipment. This research was approved by the University of Alberta’s Research Ethics Board (REB) that also meets the requirements of Canada’s Tri-Council Policy Statement for the Ethical Conduct of Research Involving Humans and all subjects provided their written consent and voluntary agreed to participate. Afterwards, the rowers were also coached on the extent to which they would be required to extend and lean back (LB) at the finish of the rowing stroke and were asked to practice this technique during their regular training sessions prior to testing to reduce any learning effect ( Picture 1 ). The schedule required a separate day for an aerobic fitness assessment (V̇O _2_ peak ) and different day for the 3 experimental trials that were performed in a random order. All rowing testing was performed on a Concept IID rowing machine (Morrisville, Vermont) and each of the 3 experimental trials consisted of a steady-state, graded exercise of increasing intensity:

- Trial 1. Rowing at 3 different power outputs (PO) equal to 125, 150 and 175 W with a corresponding stroke rate (SR) of 18, 22 and 24 strokes×min ^−1^ , respectively while using a more upright position at the finish ( Picture 1 ). This trial was considered to be the control condition.
- Trial 2. Rowing at the same PO’s as in Trial 1 (125, 150 and 175 W) using a greater LB position at the finish ( Picture 1 ). Stroke rate was not intentionally controlled since this trial was intended to match the PO used in Trial 1 and determine the effect of a greater LB position on changes in SR, as well as kinematic and physiological responses.
- Trial 3. Rowing at the same 3 SR’s as in Trial 1 (18, 22 and 24 strokes×min ^−1^ ) but with the same greater LB position used in Trial 2. PO was not controlled since this trial was intended to determine the effect of the greater LB position on changes in PO as well as kinematic and physiological responses.

This experimental design allowed for a comparison of the two different lean back rowing positions while exercising at the same power output as well as at the same stroke rate. Note that the participants were instructed to avoid any changes in the other phases of the rowing stroke during all experimental trials.

### Statistical Analysis

All group data is expressed as means and standard deviations. Gross exercise efficiency was calculated at each exercise intensity during all 3 experimental trials as the amount of work done during rowing exercise ÷ total energy expenditure × 100. Separate 2-way analysis of variance (ANOVA) procedures with repeated measures were used to compare the 3 different intensities of exercise between the 3 different experimental conditions for all dependent variables. Significant F ratios were further examined with a Newman Kuels multiple comparison procedure and alpha was preset at p < 0.05 for all analyses. Note due to the small sample size of the women, no gender comparisons were made.

## Results

Power output, HR, V̇O _2_ and energy expenditure were significantly higher when the greater LB position at the finish of the rowing stroke was performed at the same SR in comparison to the upright body position and when compared to the greater LB position at the same PO as the upright position ( Table 1 ). Stroke rate was significantly lower when the greater LB position was executed at the same PO in comparison to the other two trials. There was a linear increase in PO, SR HR, V̇O _2_ and energy expenditure across all three exercise intensities regardless of the LB position at the finish (p<0.05). The 3 intensities of rowing used in each experimental trial were performed at a range of 55–71, 69–82 and 78–91% of peak VO _2_ , respectively. Gross percent efficiency was greatest during the highest submaximal exercise intensity in comparison to the 2 lower exercise intensities (p<0.05). The % efficiency was highest when the greater LB position at the same SR was used at all 3 exercise intensities.

There was a significant larger ROM at the ankle with the greater LB position at the finish using the same stroke rate in comparison to the upright position ( Table 1 ). The greater ROM at the ankle was primarily due to a smaller ankle angle at the catch (p<0.05) since there was no significant differences in the angle of the ankle at the finish ( Table 2 ). The ROM about the ankle was significantly less during the low intensity, submaximal exercise trial compared to the two higher exercise intensities regardless of LB position.

Knee angle ROM significantly increased across all 3 exercise intensities and was greatest during the highest exercise intensity. This was primarily the result of greater knee flexion at the catch position (p<0.05). Also, the knee joint angle at the catch was smaller during the greater LB position when performed at the same stroke rate compared to the upright position (p<0.05). Knee angle at the finish was smaller with the greater LB positions and during the highest exercise intensity compared to the upright position (p<0.05). Elbow angle ROM was greatest during the two LB trials versus the upright trial (p<0.05). Elbow angle at the finish was larger during the upright trial compared to LB trials (p<0.05).

Hip angle at the finish was significantly greater during the trials in which the participants leaned farther back at the finish compared to the more upright position. Hip angle ROM was significantly higher during the two trials of greater LB position in comparison to the upright position (p<0.05). This corresponded to a significantly greater hip angle at the finish with no change at the catch (p<0.05).

There was a significant increase in the distance the handle of the rowing machine travelled from the catch position to the finish when the greater LB technique was used ( Table 1 ). There were no differences in height of the handle when moved from catch to finish between trials or intensities of exercise. Performing the greater LB position at the same PO as the upright position resulted in the longest time for handle movement between the catch and finish positions compared to the other two trials whereas the greater LB at the same SR required more time compared to the upright trial (p<0.05). As well the time required to move the handle from the catch to the finish significantly shortened as exercise intensity increased. The velocity at which the handle moved from the catch to the finish position was fastest with the greater LB trial performed at the same SR as the upright at all 3 exercise intensities (p<0.05). There was a significant increase in handle velocity as exercise intensity increased in all three trials.

## Discussion

This study examined the physiological and kinematic differences of stationary rowing exercise using two different body positions at the finish of the rowing stroke; a common, more upright posture versus a greater lean back posture. Furthermore, these two different techniques were compared at the same rowing power output, at the same stroke rate and at three different exercise intensities. As was hypothesized, rowing with a greater lean back position required higher oxygen consumption, heart rate and therefore, higher energy expenditure at all three exercise intensities in comparison to rowing with a more upright rowing posture when the same stroke rate was performed. Interestingly, exercise efficiency was greatest with the greater lean back posture at the same stroke rate in comparison to both other trials especially at the highest exercise intensity. Kinematically, the greater lean back rowing posture resulted in a larger hip, ankle and elbow range of motion. The handle of the rowing machine traveled a longer distance during a stroke and at a higher velocity when the greater LB posture was performed; however, there was no change in handle vertical excursion during the stroke. These differences suggest that a greater lean back position at the finish of the stroke, despite requiring greater energy expenditure, produces a greater power output and therefore faster performance time with no kinematic disadvantage during submaximal stationary rowing exercise.

The physiological requirements of different types of exercise depend on a variety of factors ( Zeni et al., 1996 ) and stationary rowing exercise is somewhat unique in that the amount of power output generated is influenced by the stroke rate and the amount of force applied to the handle of the machine as a result of leg, hip and torso extension and elbow flexion performed in a coordinated fashion ( Bompa, 1980 ; Martindale and Robertson, 1984 ; Torres-Moreno et al., 1999 ; Barrett and Manning, 2004 ; Webster et al., 2006 ). Our lab has shown that rowing training can influence kinematic changes and different physiological adaptations can result from training at different stroke rates ( Bell et al., 1998 ; Webster et al., 2006 ). One factor that can have a direct positive impact on increasing the amount of power output generated at a particular stroke rate is the extent of the lean back at the finish of the drive phase of the rowing stroke. This was certainly the case in the present study, as the greater lean back position at the finished produced a significant greater power output of ∼18% at all intensities of exercise combined when compared to the more upright rowing position at the same stroke rate. It was also hypothesized that the physiological consequence of a greater extension about the hip during rowing would be a greater energy demand due to the increase in muscular work. The present study confirmed this and showed that a difference of ∼28 degrees in the angle of the hip at the finish of the rowing stroke produced a significant increase in heart rate and oxygen uptake, resulting in an ∼15% higher exercise energy expenditure when rowing at the same stroke rate when all three exercise intensities were combined. When the greater lean back position was compared to the upright posture at the finish using the same power output, there were no physiological differences observed other than a lower stroke rate was required to match the power output. These data provide evidence that modifying the body position to increase the extent that an individual extends at the hip and leans further back at the finish during stationary rowing, produces a greater power output but at a higher energy expenditure at three different submaximal exercise intensities. Furthermore, the relative gain in power output would translate into a decreased time to complete a given distance during stationary rowing exercise.

The increase in energy expenditure using the greater lean back position at the finish could be viewed somewhat negatively, as greater energy expenditure may lead to an earlier onset of fatigue during exercise. The extent of changes in kinematics associated with fatigue during rowing training sessions is controversial ( Holt et al., 2003 ; Mackenzie et al., 2008 ). Interestingly, the present study showed that efficiency (a relative increase of ∼6% with the greater LB position combining all 3 exercise intensities) was significantly enhanced when the greater lean back position was performed in comparison to the more upright posture at the same stroke rate. Furthermore, exercise efficiency was greatest during the highest intensity of rowing exercise regardless of the body position used. This latter finding is supported by Di Prampero et al. (1971) who observed greater % efficiency with higher intensities of rowing exercise. Thus, the higher energy expenditure required while rowing at the same stroke rate with a greater lean back position, was partially off-set by an improvement in efficiency. This higher power output coupled with the improved efficiency may outweigh the relative increase in energy expenditure and any consequential fatigue aspects depending on the distance rowed. Thus, the present data would suggest that the energy cost to power output benefit would support using a greater lean back at the same stroke rate if a greater power output and therefore improved performance time was desired during stationary submaximal rowing exercise.

The primary difference of the two different lean back positions was the hip angle at the finish that resulted in a greater ROM about the hip at the same stroke rate and at the same power output when compared to upright position. However, the requirement of this greater hip angle may have influenced other joint angles and the present study found this to be the case. The greater lean back position produced a greater ROM about the ankle and that was primarily the result of changes in the angle of the ankle at the catch position. In other words, when the participants recovered to the catch position after completion of the greater lean back position, the shins were in a more vertical position. This can put the rower at a disadvantage during initial leg and hip power development if this change in ankle angle resulted in the shin moving past the vertical at the catch ( Nolte, 2005 ). However, the difference was only ∼3° in the ankle angle and the shin did not go past the vertical position in the present study so this should not have been a disadvantage. The higher ROM at the elbow during both greater lean back trials was due to a greater flexion at the finish compared to the upright position. The optimal position of the elbow at the finish of the stroke is somewhat controversial (Bompa, 1990). There were some changes in the angle of the knee at the catch and finish when leaning back further at the finish that offset each other since there were no significant differences in the ROM about the knee between trials despite a greater ROM with increasing intensity of exercise. Other than the anticipated differences in the angle of the hip at the finish and the hip ROM between the two body positions at the finish, the subsequent changes in the ankle, knee and elbow angles ranged from 2–4 degrees and although significant, could not be considered to have negative consequences to stationary rowing technique. This is especially in light of the large increases in power output and improved rowing exercise efficiency observed in the present study.

Another anticipated consequence of a greater lean back position at the finish is that distance the handle travels is increased in comparison to the upright posture at the finish regardless of stroke rate or power output generated. At the same stroke rate, this translates into a greater handle velocity and subsequent power output as shown in the present study. Handle movement can have a negative impact on rowing mechanics and application of force ( Torres-Moreno et al., 1999 ), but in the present study, the height of the handle as it moved through the drive phase was not significantly different between trials. This provides further evidence that there was little negative consequence to the direction the handle moved during stationary rowing when a greater lean back position was performed.

In conclusion, rowing exercise performed on stationary machines is used for general conditioning by recreational individuals, for supplementary training for rowers, and for performance testing and competition ( Klusiewicz et al., 1999 ; Gillies and Bell, 2000; C.R.A.S.H.-B Sprints, 2012 ; Concept II, 2012). Thus, any change in technique of rowing on a rowing machine that can improve performance would be of great interest to these individuals. The present study showed that rowing with a greater lean back position as evidenced by a higher ROM and angle of hip at the finish of the rowing stroke produced a significant increase in power output at the same stroke rate regardless of the intensity of the exercise in comparison to a more upright body position. However, this occurred at a higher metabolic cost which may have implications for fatigue development. Despite this, the greater relative increase on PO coupled with a significant improvement in the efficiency of stationary, submaximal rowing exercise when leaning back further at the finish and no suggested negative kinematic changes, we would advocate that the gains in power output outweigh the greater energy required. The greater lean back at the finish may also be a method of increasing exercise intensity during training sessions if desired by the athlete or the coach that may translate into improved fitness. It remains to be determined to what extent a greater lean back at the finish of a rowing stroke can influence on water rowing speed.

## Figures and Tables

**Picture 1:**
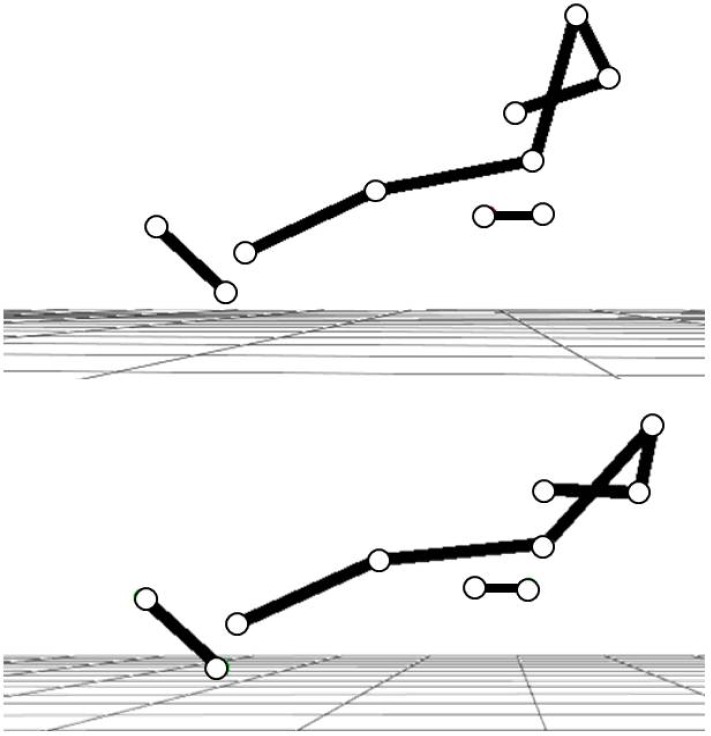
*
The two different lean back positions at the finish of a rowing stroke used in the present study.
* *
The top panel represents the upright position and the bottom panel represents the greater LB position.
*

**Table 1:** *
The effect of different lean back positions at the finish of the rowing stroke on power output, stroke rate, various physiological parameters, joint range of motion and characteristics of the handle movement at 3 different intensities of exercise on a Concept IID rowing machine. Values are means ± SD* .

Variable	Exercise Intensity 1			Exercise Intensity 2			Exercise Intensity 3		
	Upright	LB @Same PO	LB @ Same SR	Upright	LB @ Same PO	LB @ Same SR	Upright	LB @ Same PO	LB @ Same SR
PO (w)	125 ±3	127 ±3	156 ±21 ^a , b^	157 ±22 ^c^	151 ±3 ^c^	184 ±31 ^a , b , c^	174 ±3 ^d^	175 ±2 ^d^	216 ±38 ^a , b , d^
SR (strokes ×min ^ −1 ^ )	18 ±1	17 ±3 ^a^	19 ±1 ^b^	22 ±1 ^c^	20 ±2 ^a , c^	22 ±1 ^b , c^	24 ±1 ^d^	21 ±3 ^a , d^	25 ±1 ^b , d^
HR (beats ×min ^ −1 ^ )	146 ±21	151 ±21	158 ±20 ^a , b^	157 ±22 ^c^	161 ±22 ^c^	172 ±18 ^a , b , c^	168 ±24 ^d^	170 ±24 ^d^	183 ±16 ^a , b , d^
V̇O _ 2 _ (L×min ^ −1 ^ )	2.13 ±0.13	2.43 ±0.21	2.71 ±0.48 ^a , b^	2.65 ±0.11 ^c^	2.75 ±0.20 ^c^	3.15 ±0.54 ^a , b , c^	3.01 ±0.15 ^d^	3.05 ±0.21 ^d^	3.48 ±0.57 ^a , b , d^
Energy Expenditur e(kJ×min ^ −1 ^ )	47.7 ±2.7	49.4 ±4.2	55.7 ±10.2 ^a , b^	55.1 ±2.2 ^c^	56.7 ±3.9 ^c^	65.7 ±11.7 ^a , b , c^	63.1 ±3.0 ^d^	63.6 ±4.22 ^d^	73.5 ±12.2 ^a , b , d^
% Gross Exercise Efficiency	15.8 ±1.1	15.5 ±1.3	17.2 ±3.3 ^a , b^	16.4 ±0.7	16.1 ±1.1	16.8 ±0.9 ^a , b^	16.6 ±0.7 ^d^	16.6 ±1.1 ^d^	17.6 ±0.9 ^a , b , d^
Ankle ROM (°)	77 ±6	79 ±5	80 ±5 ^a^	78 ±6 ^e^	80 ±5 ^e^	82 ±5 ^a , e^	79 ±8 ^e^	81 ±13 ^e^	83 ±4 ^a , e^
Knee ROM (°)	112 ±6	113 ±7	114 ±7	114 ±6 ^e^	114 ±7 ^e^	115 ±6 ^e^	116 ±5 ^e^	116 ±3 ^e^	116 ±6 ^e^
Hip ROM (°)	85 ±7	113 ±9 ^a^	113 ±8 ^a^	85 ±8	113 ±9 ^a^	111 ±9 ^a^	83 ±8	116 ±8 ^a^	111 ±10 ^a^
Elbow ROM (°)	96 ±12	101 ±14 ^a^	103 ±13 ^a^	94 ±13	101 ±13 ^a^	101 ±14 ^a^	92 ±13	103 ±9 ^a^	101 ±14 ^a^
Handle Distance (m)	1.37 ±0.14	1.55 ±0.16 ^a^	1.56 ±0.16 ^a^	1.38 ±0.13	1.55 ±0.17 ^a^	1.55 ±0.17 ^a^	1.38 ±0.12	155 ±0.15 ^a^	1.56 ±0.15 ^a^
Handle Height (m)	0.12 ±0.10	0.13 ±0.07	0.14 ±0.07	0.11 ±0.09	0.12 ±0.07	0.13 ±0.06	0.11 ±0.09	0.13 ±0.08	0.13 ±0.06
Handle Time Per Stroke (s)	1.13 ±0.11	1.25 ±0.13 ^a^	1.18 ±0.10 ^a , b , d^	1.06 ±0.09 ^c^	1.18 ±0.12 ^a , c^	1.11 ±0.09 ^a , b , c , d^	1.01 ±0.07 ^c^	1.12 ±0.10 ^a , c^	1.03 ±0.06 ^a , b , c , d^
Handle Velocity Per Stroke (m×s ^ −1 ^ )	1.22 ±0.08	1.24 ±0.08	1.32 ±0.11 ^a , b^	1.30 ±0.08 ^c^	1.32 ±0.06 ^c^	1.39 ±0.12 ^a , b , c^	1.37 ±0.07 ^c , d^	1.39 ±0.05 ^c , d^	1.51 ±0.12 ^a , b , c , d^

*LB = lean back, PO = power output, SR = stroke rate, HR = heart rate,*

V̇O _2_ *= volume of oxygen consumed per min, ROM = range of motion.*

*a = significantly different from the upright trial, main effect, P<0.05.*

*b = significantly different from the lean back trial at the same PO, main effect, P<0.05.*

*c = significantly different from exercise intensity 1, main effect, P<0.05.*

*d = significantly different from exercise intensity 1 and 2, main effect, P<0.05.*

*e = significantly different from exercise intensity 2, main effect, P<0.05.*

**Table 2:** *
The effect of different lean back positions at the finish of the rowing stroke on ankle, knee, hip and elbow angles at the catch and finish at 3 different intensities of exercise on a Concept IID rowing machine. Values are means ± SD.
*

Exercise Intensity 1						
	Upright		LB @ Same PO		LB @ Same SR	

Joint Angle	Catch	Finish	Catch	Finish	Catch	Finish
Ankle (°)	35±6	112±3	33±6	112±2	32±6 ^a^	112±3
Knee (°)	52±7	164±6	49±7	162±4 ^d^	49±7 ^a^	162±3 ^d^
Hip (°)	33±7	118±4	32±7	145±5 ^d^	32±7	145±5 ^d^
Elbow (°)	163±6	68±12	161±7	60±14 ^d^	161±7	58±14 ^d^

Exercise Intensity 2						
	Upright		LB @ Same PO		LB @ Same SR	

Joint Angle	Catch	Finish	Catch	Finish	Catch	Finish
Ankle (°)	34±6 ^b^	112±3	31±6 ^b^	112±3	30±6 ^a , b^	112±3
Knee (°)	50±7 ^b^	164±5	48±7 ^b^	162±4 ^d^	45±7 ^a , b^	151±4 ^d^
Hip (°)	32±7	117±6	32±7	145±5 ^d^	32±7	143±5 ^d^
Elbow (°)	162±7	68±13	161±7	59±14 ^d^	161±8	60±14 ^d^

Exercise Intensity 3						
	Upright		LB @ Same PO		LB @ Same SR	

Joint Angle	Catch	Finish	Catch	Finish	Catch	Finish
Ankle (°)	32±7 ^c^	111±4	30±6 ^c^	112±3	29±6 ^a , c^	112±3
Knee (°)	47±8 ^c^	163±5 ^e^	45±8 ^c^	161±4 ^d , e^	44±8 ^a , c^	160±4 ^d , e^
Hip (°)	33±7	116±5	31±7	144±4 ^d^	31±7	143±6 ^d^
Elbow (°)	161±6	69±14	160±7	61±13 ^d^	159±7	59±13 ^d^

*LB = lean back, PO = power output, SR = stroke rate.*

*a = significantly different from the upright trial at the catch, main effect, P<0.05.*

*b = significantly different from exercise intensity 1 at the catch, main effect, P<0.05.*

*c = significantly different from exercise intensity 2 at the catch, main effect, P<0.05.*

*d = significantly different from the upright trial at the finish, main effect, P<0.05.*

*e = significantly different from exercise intensity 1 at the finish, main effect, P<0.05.*
